# Voluntary physical activity and pharmacological treatment with the Ginkgo biloba extract EGb 761 alleviate regional alterations in cerebral synaptic density and improve behavioral recovery after unilateral labyrinthectomy in the rat

**DOI:** 10.3389/fneur.2026.1937612

**Published:** 2026-10-23

**Authors:** Isabelle S. Fuxjäger, Magdalena J. Lindner, Gunnar P. H. Dietz, Rosel Oos, Giovanna Palumbo, Anja Krämer, Christina Branner, Astrid Delker, Maximilian Grosch, Simon Lindner, Karin Wind-Mark, Sibylle I. Ziegler, Peter Bartenstein, Matthias Brendel, Rudolf A. Werner, Melissa J. Antons, Andreas Zwergal

**Affiliations:** 1 German Center for Vertigo and Balance Disorders (DSGZ), LMU University Hospital, LMU Medizin, LMU Munich, Munich, Germany; 2 Department of Global Medical Affairs, Dr. Willmar Schwabe GmbH & Co. KG, Karlsruhe, Germany; 3 Department of Neurology, University Medical Center Göttingen, Göttingen, Germany; 4 Department of Nuclear Medicine, LMU University Hospital, LMU Medizin, LMU Munich, Munich, Germany; 5 Clinic for Nuclear Medicine, Hannover Medical School (MHH), Hannover, Germany; 6 German Center for Neurodegenerative Diseases (DZNE), Munich, Germany; 7 Munich Cluster for Systems Neurology (SyNergy), Munich, Germany; 8 Department of Radiology, LMU University Hospital, LMU Medizin, LMU Munich, Munich, Germany; 9 Department of Neurology, LMU University Hospital, LMU Medizin, LMU Munich, Munich, Germany

**Keywords:** acute vestibular syndrome, ginkgo biloba, neuroplasticity, vestibular compensation, vestibular rehabilitation

## Abstract

**Background:**

Unilateral peripheral vestibular loss induces adaptive mechanisms of cerebral plasticity. The quantified Ginkgo biloba extract EGb 761^®^ has been shown to enhance neural plasticity, and is used in the treatment of vertigo and dizziness. We here aimed to investigate, how voluntary physical training in a running wheel (RWT) and oral treatment with EGb 761^®^ affect behavioral recovery and whole-brain synaptic density following chemical unilateral labyrinthectomy (cUL) in the rat.

**Methods:**

Thirty-two Sprague Dawley rats were divided into 4 groups with different treatment regimens (group 1: control food; group 2: control food + RWT; group 3: EGb 761^®^ food (75mg/day/kg body weight); group 4: EGb 761^®^ food + RWT). All rats underwent a left-sided cUL by transtympanic injection of bupivacaine and p-arsanilic acid. Behavioral testing (scoring of nystagmus, postural asymmetry, head tilt, locomotion tracking in open field) was done weekly until 9 weeks (d63) after cUL. Serial [^18^F]UCB-H μPET imaging of synaptic density (targeting synaptic vesicular protein 2A) was performed before and up to 9 weeks post cUL. Images were whole-brain normalized and segmented based on Schiffer's atlas. Voxel-wise and regional changes of cerebral [^18^F]UCB-H uptake were compared across groups and time points.

**Results:**

All rats showed signs of acute vestibular syndrome following cUL. The EGb 761^®^ + RWT group had a significantly faster and more complete improvement of postural asymmetry by week 5 post UL and a higher locomotor velocity thereafter than the control food group. Compared to baseline, at d63 post cUL the EGb 761^®^ group showed the smalles reduction of synaptic density in the vestibular nuclei of−7.59 ± 7.0 % (vs.−20.37 ± 5.18% in control food group, *p* < 0.001). In contrast, at d63 post cUL the motor cortex displayed the highest relative gain of synaptic density in the control food group (6.6 ± 3.13 % vs. 0.19 ± 2.81% in the EGb 761^®^ group, *p* < 0.001). All treatment groups showed an increase of synaptic density in the hippocampal area and the somatosensory cortex at weeks 1-3 post cUL compared to the control food group. The additive effect of EGb 761^®^ and RWT was statistically confirmed in behavior and PET measurements.

**Conclusions:**

Unilateral labyrinthectomy induces loss of synaptic density in vestibulo-cerebellar networks, which can be partially reversed by EGb 761^®^ treatment. Combined physical and EGb 761^®^ treatment improves behavioral recovery from cUL most effectively, reinforcing the importance of multimodal treatment in acute vestibulopathies.

## Introduction

Acute unilateral vestibular loss leads to symtoms of vestibular tone asymmetry such as vertigo, oscillopsia, falling tendency or gait ataxia, which subsequently recover in a process of central vestibular compensation driven by adaptive lesion-induced neuroplasticity (1, 2). Modern treatment concepts for acute vestibular loss aim to foster central vestibular compensation through early and intense vestibular physical therapy (3). While the evidence for a therapeutic benefit of vestibular physical therapy in unilateral vestibular loss is abundant, the underlying neurobiological mechanisms are less clear. In addition, the effects of training can be augmented by the application of pharmaceutical agents, which enhance cerebral neuroplasticity, such as the quantified polyvalent Ginkgo biloba extract EGb 761^®^ (4–9).

EGb 761^®^ is composed of various pharmacologically active components, including ginkgolides and terpenlactones (10). Previous studies in various animal models have consistently shown a significant acceleration of functional recovery from unilateral vestibular loss by EGb 761^®^ (6, 7, 9). Recently, it was found that this dose-dependent therapeutic effect is conveyed by ginkgolide A and B and partially accomplished via a change of cerebral glucose metabolism in central vestibular network hubs (7). In addition, a faster synaptic reoccupation in the deafferented vestibular nucleus with EGb 761^®^ treatment was described by histological analysis as a potential long-term mechanism of structural vestibular compensation (6). Indeed, the pharmacological actions of EGb 761^®^ and its constituents on neuroplasticity have been examined in various model systems: The Ginkgo extract constituent bilobalide increases the efficiency of synaptic transmission in the hippocampus and improves its plasticity (11). EGb 761^®^ and its constituents protect and promote neuronal cell connectivity. EGb 761^®^ dose-dependently increases the division rate of hippocampal neurons (12). Standardized Ginkgo extract (13, 14) Ginkgolide B (15), Isorhamnetin (16), Quercetin (17–20) and bilobalide promote plasticity, sprouting, growth, or regeneration of neural processes and protect synapses from damage by ß-amyloid (21, 22). There are also scientific studies on the molecular mechanism of the effect of EGb 761^®^ on nerve cell connectivity. For example, EGb 761^®^ is known to restore the Aβ-oligomer inhibited phosphorylation of cAMP response element (CREB) to normal (22). CREB, in turn, plays an important role in the regulation of neuronal plasticity, i.e. the adaptive capacity of the brain. These mechanisms may also explain the preventive effects of EGb 761^®^ in neurocognitive disorders such as mild cognitive impairment or dementia, which were substantiated by high-level evidence from multiple randomized controlled trials (23). Meta-analysis furtheron indicated that also accompanying sensory complaints in patients with neurocognitive disorders such as dizziness or tinnitus improved under EGb 761^®^ relative to placebo treatment (24).

The present four-arm study aimed to directly compare the effects of voluntary physical exercise with running wheel training (RWT), pharmacological treatment with EGb 761^®^ and a combination of both against a control condition on behavioral recovery from unilateral labyrinthectomy in the rat and to analyse differences in longitudinal whole-brain synaptic density using serial *in vivo* synaptic vesicle protein 2A (SV2A) positron emission tomography across groups. This design was inspired by previous studies, which showed effects of physical exercise and EGb 761^®^ treatment on synaptic plasticity by immunohistochemistry and the clinical experience that a combined physical and pharmacological treatment may have overadditive benefits against either treatment alone in patients with peripheral vestibular deficits. We hypothesized that 1) EGb 761^®^ and voluntary RWT will independently enhance vestibular compensation by augmentation of synaptic reoccupation in the deafferented central vestibular hubs, as well as synaptic plasticity in cerebral sensorimotor networks, and 2) combined intervention will have superior effects on behavioral recovery from vestibular asymmetry. Essentially, the integrative approach of the current study strives to elucidate whether combined physical and pharmacological interventions targeting adaptive lesion-induced neuroplasticity, can enhance both functional recovery and synaptic plasticity following unilateral vestibular loss.

## Methods

### Animals

All animal experiments were approved by the government of Upper Bavaria and performed in accordance with the guidelines for the use of living animals in scientific studies and the German Law for the Protection of Animals (license number 55.2-1-54-2532-93-16). Thirty-two male Sprague-Dawley rats (mean 400 ± 20 g, age 3 months at time of chemical unilateral labyrinthectomy, cUL, Charles River Ltd, UK) were housed two animals per cage in a temperature and humidity-controlled room with a 12 h light/dark cycle, with free access to food and water.

### Experimental design

To test the effect of voluntary physical training with RWT, EGb 761^®^ and the combination of both on vestibular compensation, 32 rats were divided into 4 groups. Group 1 and 2 received control food throughout the experiment. Group 1 (*n* = 8) was kept in standard rat IVC cages (Tecniplast, GR1800 double Decker), while group 2 (*n* = 8) was placed in a cage system with a running wheel (Tecniplast, Intellibio) after the baseline measurement. Group 3 and 4 received EGb 761^®^ food (75 mg/day/kg body weight) throughout the experiment. Group 3 (*n* = 8) was housed in the rat IVC cages, group 4 (*n* = 8) in cage systems with running wheels. Subsequent behavioral scoring was performed at baseline and day 1, 2, 3, 5, 7 and then weekly until week 9 post cUL in all rats. To quantify the effects of therapeutic interventions on whole-brain synaptic density *in vivo* after cUL, μPET measurements with the radiotracer [^18^F]UCB-H were done in rats of all groups at baseline, week 1, 3 and 9 post cUL (Figure 1) as binding of this tracer to synaptic vesicle protein 2A (SV2A) has been shown to serve as surrogate measure of synaptic density (25–28). On days with μPET recording, behavioral scoring was accomplished before scanning.

**Figure 1:**
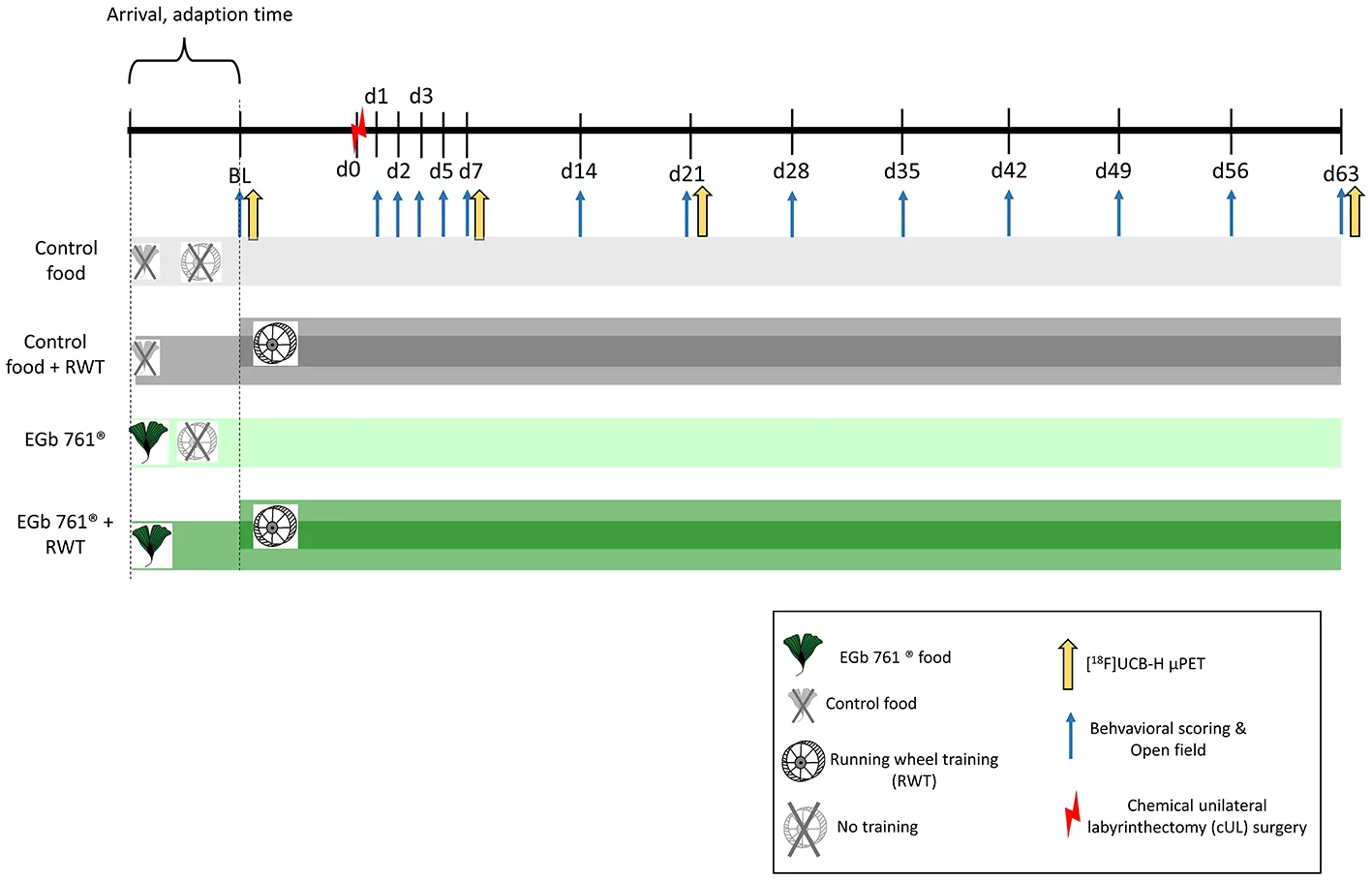
Study design and time course of treatment, behavioral testing and μPET imaging. After a habituation period of 6 weeks, where rats received either control food (groups 1 and 2, *n* = 8 each) or EGb 761^®^ food (groups 3 and 4, *n* = 8 each), baseline [^18^F]UCB-H μPET and behavioral testing was carried out. Thereafter, groups 2 and 4 were placed in the running wheel cages. After chemical unilateral labyrinthectomy (cUL), rats underwent behavioral testing and [^18^F]UCB-H μPETs at defined sequential postoperative time points as indicated. At the end of the experiment (d63 post cUL) the animals were perfused and their brains collected.

### Therapeutic interventions

#### Running wheel training

During the 6-weeks habituation period, all animals were kept in a double-decker rat cage (GR1800 Double decker for rats, Tecniplast). After baseline imaging, a microchip was implanted subcutaneously between the shoulder blades of all animals under general isoflurane anesthesia (described in section about unilateral chemical labyrinthectomy). The running wheel groups (group 2 and 4) were put into cage systems from Intellibio in pairs for 3 days before cUL to get used to the novel environment. Each individual cage was equipped with a running wheel that the rats could use voluntarily. The individual acceptance, the running interval and the distance covered was determined for each animal by detection via the implanted microchip. Wheel turns were automatically monitored by computer and stored at 360-s intervals for each wheel by ActivityWheel software (Intellibio, Seichamps, France).

#### Pharmacological treatment with EGb 761^®^

Phytoestrogen-free food with Ginkgo biloba extract EGb 761^®^ supplement and without EGb 761^®^ supplement (control food) was provided by Altromin Spezialfutter GmbH & Co. KG, Germany. EGb 761^®^ was supplied by Dr. Willmar Schwabe Pharmaceuticals, Karlsruhe, Germany. The dry extract is extracted from Ginkgo biloba leaves (35–67:1) by using 60% acetone. The extract is adjusted to 22–27% Ginkgo flavonoids calculated as Ginkgo flavone glycosides, 5–7% terpene lactones consisting of 2.8–3.4% ginkgolides A, B, C, and 2.6–3.2% bilobalide and contains < 5 ppm ginkgolic acids (EGb 761^®^ is the active ingredient of Tebonin^®^ intens, Dr. Willmar Schwabe Pharmaceuticals, Karlsruhe, Germany). The EGb 761^®^ food contained 75 mg/day/kg body weight of this quantified Ginkgo biloba extract following the treatment regimen of a previous study, which showed an acceleration of vestibular compensation with this dosage (7). Food was prepared in 10 mm palletization and color-coded to avoid carry-over or mix-up (EGb 761^®^ food: yellow, control food: green). Animals were allowed to consume the food *ad libitum*. Food was exchanged weekly and the extent of food consumption per cage was documented. Feeding with EGb 761^®^ or control food was started 6 weeks prior to cUL to allow for habituation and established pharmacological action at the time of chemical unilateral labyrinthectomy.

### Chemical unilateral labyrinthectomy

Chemical unilateral labyrinthectomy (cUL) was performed as described previously (4, 29, 30). Animals were anesthetized with 1.5 % isoflurane in O2 delivered up to 1.2 l/min O2 via a mask. For surgical analgesia, 1.5 mg/kg meloxicam was injected subcutanously 30 min before and 3 days after surgery. An additional 5 ml saline was injected subcutanously as a bolus to maintain fluid balance. After local anesthesia with 1 % bupivacaine hydrochloride, a left paramedian incision was made to expose the lamboidal ridge and the external ear canal. The external ear canal was opened just anterior to the exit point of the facial nerve. With a 26-gauge needle the tympanic membrane was perforated caudally to the hammer shaft, and 150 μl of a 20 % bupivacaine solution was injected into the tympanic cavity. After 2 min the bupivacaine solution was aspirated and instilled again slowly (three repetitions in total). The same procedure was repeated with 150 μl of 10 % solution of p-arsanilic acid, which irreversibly desensitized the primary sensory cells of the inner ear. After the last thorough aspiration, the wound was closed by skin suture and for preventive antibiosis 2 mg/kg marbofloxacin was injected subcutanously for 3 days.

### Criteria for exclusion

Animals were excluded from the study if the following symptoms were observed:

- Loss of body weight exceeding 20% of the pre-treatment value.
- Ulcer of the cornea, which could occur due to an inadvertent lesion of the facial nerve during cUL.
- Bleeding from the tympanic cavity, which could impair the diffusion of bupivacaine or p-arsanilic acid into the inner ear.
- Abnormalities in behavioral scoring, i.e., convulsions, paresis, hemiataxia, etc.

Based on these criteria, no animals were excluded from the study. However, two animals died during the course of the experiment (one from the EGb 761^®^ group, intraoperatively; the other from the Control food group, at day 63). Data from these animals were included up to the time of death.

### Clinical behavioral scoring of vestibular asymmetry after cUL

Behavioral symptoms of vestibular asymmetry were scored by two experienced veterinarians blinded for the treatment applying an established scoring system (31): nystagmus, postural asymmetry, head roll tilt, and elevation tail test. Scores were depicted as mean values of two raters. Each component was given a score of up to 10:

- *Nystagmus* was observed visually. Intensity of spontaneous nystagmus was scored with 6–10 points, with 1 point for every 60 beats per minute (bpm). In the absence of spontaneous nystagmus at rest, the animal was touched slightly. If nystagmus was evoked, it was scored 1–5 points, with 1 point for every 60 bpm.
- *Postural asymmetry* was scored as follows: spontaneous barrel rolling−10 points; barrel rolling evoked by light touch or airpuff−9 points; recumbent position on lesion side without leg support−8 points; some ipsilesional leg support−7 points; moving around on one side or using ipsilesional legs for recumbent support−6 points; moving around with bilateral leg support−5 points; moving around with occasional falls to the ipsilesional side−4 points; moving around leaning toward the ipsilesional side−3 points; hardly noticeable asymmetry−2 points; postural asymmetry only noticeable when picked up−1 point.
- Spontaneous *head roll tilt* was scored by estimating the angle between the jaw plane and the horizontal, with 10 points given either for a 90° angle or if the animal rested recumbent on the lesion side or showed barrel-rolling toward that side. Seven points correlated to a 60° and 5 points to a 45° angle.
- Influence of perceptive somatosensory input to postural control during vestibular compensation was examined by the *elevation tail rotation test*. Animals were picked up from the ground at the radix of their tail and body rotation was scored by estimating in degrees, with 10 points given for more than 360°, 8 points for 180–360°, 6 points for < 180° and 0 points for no relevant rotation.

### Instrumented locomotor analysis

Testing of locomotor behavior was performed in the open field using automated video tracking (EthoVision System, Noldus, Netherlands). Animals were allowed to move freely in the rectangular open field (side length 70 cm) over 10 min. Position of nose, body center, and tail were automatically detected by a video software. Mean locomotor velocity and cumulative duration of stay in the center of the maze were quantified. Locomotor velocity was taken as an overall estimate of mobility, the time in center zone as a measure of mobility and spatial exploration behavior.

### [^18^F]UCB-H μPET imaging

Anesthesia was induced with isoflurane (as described above) and a cannula was placed in the lateral tail vein. Rats were positioned in the Siemens Inveon P120 PET scanner (Siemens Medical Solutions, Munich, Germany) and were kept warm with a heating pad. In order to avoid head movement, the head position was fixed using a custom-made head holder. A [^18^F]UCB-H bolus (40 ± 2 MBq) was injected (diluted in 0.5 ml saline) and the scan was started dynamically. A 60-min-long emission recording was initiated followed by a 7 min transmission scan using a rotating [^57^Co] point source. Upon recovery from anesthesia the rats were returned to their home cages. Animal numbers varied across PET imaging time points due to technical scanner-related issues resulting in non-evaluable scans. The numbers of animals included in each PET imaging and behavioral analysis are summarized in Supplementary Tables 1, 2.

#### Image processing methods

The PET reconstruction procedure was an Ordered Subsets Expectation Maximization (OSEM-3D) algorithm with decay correction, scatter correction, attenuation correction, dead time correction, and sensitivity normalization (Siemens Healthineers, Erlangen, Germany). The attenuation correction was enabled by transmission measurements at the end of the μPET scan. This yielded images with 128 x 128 x 159 voxels of 0.78 x 0.78 x 0.80 mm^3^. All images represented the measured [^18^F]UCB-H tracer activity 16-60 min after injection, which was used as a surrogate for cerebral synaptic density (cSD). The brains in the images were cropped and rigidly registered into PX Rat atlas space (32) in PMOD medical image analysis software (PMOD Technologies LLC, RRID: SCR_016547, v4.004). After applying a 0.4 mm isotropic Gaussian filter, normalization to the whole brain mean activity (SUV_R_) was performed using a brain mask in atlas space in a self-written Python script to achieve comparability. Subsequently, the images were segmented into 58 brain regions using Px Rat (32) atlas and SUV_R_ values for every brain region were extracted. The results for the brain blood flow region describe a vessel and were discarded, therefore 57 brain regions remained. In addition, experienced neurologists defined regions of the vestibular nuclei situated at the intersection between pons and medulla. SUV_R_ values were extracted for the nuclei as well and included in the evaluations. Additionally, voxel-wise analyses were performed in SPM12 software (Wellcome Department of Cognitive Neurology, Great Britain). Paired *t*-tests were used to compare baseline and post-cUL measurements within the same animals, whereas two-sample *t*-tests were used for comparisons between experimental groups. Statistical thresholds of *p* < 0.001 and *p* < 0.005 (uncorrected) were applied without threshold masking or multiple-comparison correction, together with a cluster extent threshold of *k* = 20 contiguous voxels. Statistical maps are presented as *t*-value maps.

### Statistical analysis

Statistical analyses were developed with the assistance of Claude (Claude Sonnet 5, Anthropic, https://claude.ai), a generative AI tool used to support model selection, R code implementation and interpretation of diagnostic output. All statistical decisions, model outputs, and results were verified by the authors, who take full responsibility for the accuracy and validity of the reported analyses. Behavioral scores (Head Tilt, Postural Asymmetry, Nystagmus and Head Tilt) were ordinal and were analyzed separately for each domain using cumulative link mixed models (CLMM; R package ‘ordinal'; RStudio version 2026.07.01), with Food, RWT and Time (and their interactions) as fixed effects and Animal ID as a random intercept. The functional form of the time effect was selected per outcome via likelihood ratio tests comparing linear, quadratic, and categorical (early/late phase) specifications; the polynomial time model was replaced by a categorical phase model for Postural Asymmetry due to near-complete separation in the late phase. For Nystagmus, the Food x RWT and Time x Food interaction terms could not be reliably estimated due to insufficient between-group variability and were omitted from the final model. For each behavioral outcome except Nystagmus (see above), each treatment group was compared against at every assessed time point (or phase, for Postural Asymmetry) using Dunnett-type contrasts on model-estimated marginal means (R package ‘emmeans'), with an additional Holm correction applied across all time points within each outcome.

Open field parameters (mean velocity, cumulative duration in center) were analyzed separately for each outcome due to differing data distributions. Mean velocity and in-center duration were modeled using linear mixed models (LMM; R package 'lm4′/'lmerTest', RStudio version 2026.07.01) with Time, Food (Control food/ EGb 761^®^) and RWT (No RWT/RWT) and their interactions as fixed effects, and Animal ID as a random intercept; model residuals were confirmed to be approximately normally distributed (Shapiro-Wilk test, visual inspection of Q-Q plots). Significance of fixed effects was assessed via likelihood ratio tests comparing nested models. *Post-hoc* comparisons of each treatment group against Control (Control + No RWT) were performed at each of the 14 assessment time points using Dunnett-type contrasts (R package 'emmeans'; multivariate t-distribution adjustment), with an additional Holm correction applied across all time points within each outcome to account for the increased number of comparisons.

Two brain regions known to be involved in vestibular compensation were chosen to represent intratentorial (left and right vestibular nuclei) and supratentorial changes (left and right motor cortices). For calculating the relative change of synaptic density on postoperative day in comparison to baseline, the following formula was used:


$$\begin{array}{l} relative\;change\;\left(\%\right)={\left(1-\frac{individual\;SUV_{R}\;day}{individual\;SUV_{R}\;baseline}\right)}^{*}100^{*}-1 \end{array}$$ (1)

Afterwards, normality was checked using the Shapiro-Wilk test revealing a normal distribution using GraphPad Prism (version v10.1.2 for Windows GraphPad Software, San Diego, CA, USA). To test for differences regarding the treatment and timepoints, a mixed effects model (REML) with the fixed effects time, EGb 761, RWT and their interaction (Time x EGb 761, Time x RWT, EGb 761 x RWT and Time x EGb 761 x RWT) as sources of variation. The random effects included the subject, the interaction of subject and time (Subject x Time), subject and RWT (Subject x RWT) and Residuals. Tukey's multiple comparison test was applied for *post-hoc* correction and *p* < 0.05 was considered significant, indicated by asterisks in graphs.

## Results

### Clinical scoring of vestibular asymmetry

All animals showed clear clinical signs of unilateral vestibular damage after cUL (Figure 2). Postural asymmetry increased during the first postoperative days due to delayed toxicity of *p*-arsanilic acid (peak at d5 for control food group; d3 in control food + RWT, EGb 761^®^ and EGb 761^®^ + RWT groups; Figure 2A). The fixed effects Phase, Food and RWT as well as the interactions Food x RWT and Phase x Food x RWT were significant (see Supplementary Table 3). Afterwards, scores declined in all groups but at different rates. Only in the EGb 761^®^ + RWT group no clinical signs of postural asymmetry were detected from d35 post cUL, whereas all other groups had some mild residual postural asymmetry (mean scores < 4) up to d63. EGb 761^®^ + RWT differed significantly from the control food group in the early (d1-d14) and late timepoints (d21-d63, Figure 2B).

**Figure 2:**
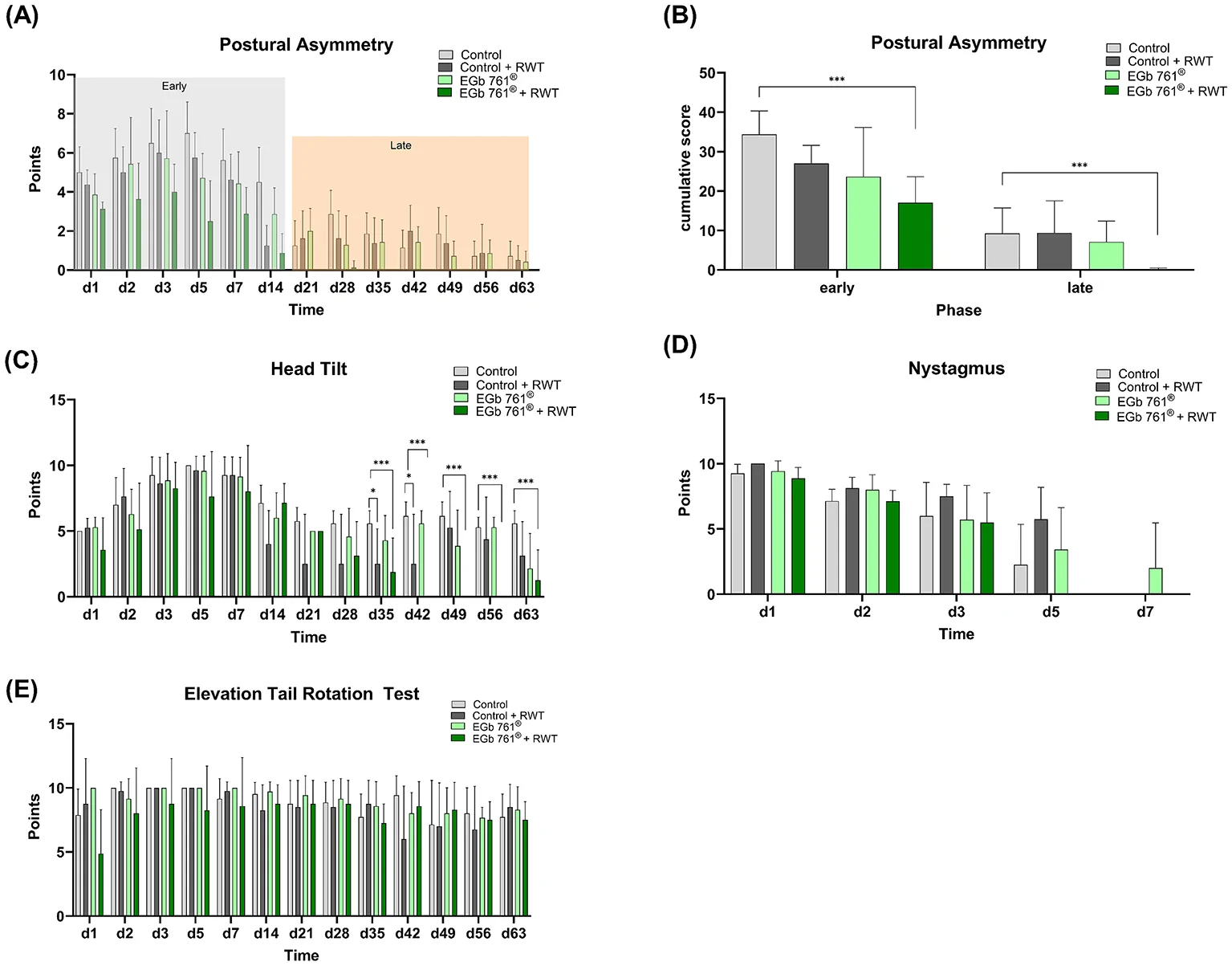
Behavioral clinical scores of vestibular asymmetry across treatment groups. Results/ scores of postural asymmetry **(A, B)**, head tilt **(C)**, nystagmus **(D)**, and elevation tail rotation test **(E)** were assessed postoperatively using cumulative link mixed models (animal as random intercept). For postural asymmetry, daily scores were summed per animal within an early (day 1-14) and a late (day 21–63) phase to obtain a cumulative score for visualization **(A, B)**, shown as group mean ± standard deviation; statistical group comparisons were based on a phase-based model (Phase x EGb 761^®^ x RWT) applied to the individual daily scores (see Methods). Head tilt and elevation tail rotation were modeled with a quadratic time term (Time^2^ x EGb 761^®^ x RWT). For nystagmus, EGb 761^®^ and RWT effects and their interactions could not be reliably estimated (see Methods); accordingly, no group-wise statistical comparisons are shown for this outcome.The values are presented as means with standard deviation. BL = baseline, control = control food, d = day, * *p* < 0.05, ** *p* < 0.01, *** *p* < 0.001.

Head tilt peaked between d5–7 in all treatment groups after cUL and improved thereafter (Figure 2C). Statistical analysis revealed a significant effect for Time, Food, RWT, Time x Food interaction, Time x RWT and Time x Food x RWT interaction (see Supplementary Table 3). Head tilt persisted until d63 in all groups except the EGb 761^®^ + RWT group, which showed no head roll tilt from d42–d56. This group had significantly lower clinical scores for head roll tilt than the control food group from d35–63. Additional significant differences were found between control food vs. control food + RWT at d35 and d42.

Nystagmus peaked at d1 post cUL in all groups (mean scores: control food: 9.3, control food + RWT: 10.0, EGb 761^®^: 9.4, EGb 761^®^ + RWT: 8.9) and resolved earliest in the EGb 761^®^ + RWT group at d5 (Figure 2D). Only the fixed effect Time had a significant effect (χ^2^ = 73.73, df = 1, *p* < 0.001). For Nystagmus, the final model retained Time, Food and RWT as fixed effects (Food x RWT and Time x Food) interaction terms could not be reliably estimated due to insufficient between-group variability (see Methods and Supplementary Table 3).

In the Elevation Tail Rotation Test, all groups showed positive responses at all postoperative time points (Figure 2E). However, only the fixed effect Time significantly influenced the score (χ^2^ = 18.75, df = 2, *p* < 0.001). Multiple comparisons revealed no significant difference between groups.

Cumulative duration spent in the running wheel markedly decreased after cUL and remained below baseline level until week 9 (with a regional peak at d14 in the control group and d21 in the EGb 761^®^) (Supplementary Figure 1). Mean values of cumulative duration in the running wheel were not significantly different across groups.

### Quantitative locomotor parameters in open field testing

For the mean velocity, the factors Time, Food and the interactions Time x Food and Time x RWT had a significant impact on locomotion speed (see Supplementary Table 4). At d1 after cUL, mean locomotor velocity and cumulative duration of movement decreased in all groups compared to baseline (Figure 3A). Over time, both parameters increased in all groups and were above baseline level after d7 post cUL. From d28, locomotor velocity was highest in EGb 761^®^ + RWT group (with a mean level of 14 cm/sec), followed by EGb 761^®^ (12.5 cm/sec), control food + RWT (11 cm/sec), and control food (9 cm/sec) groups. Rats in the EGb 761^®^ + RWT group had a significantly higher locomotor velocity than those of the control food group at d35–63 (Figure 3A).

**Figure 3:**
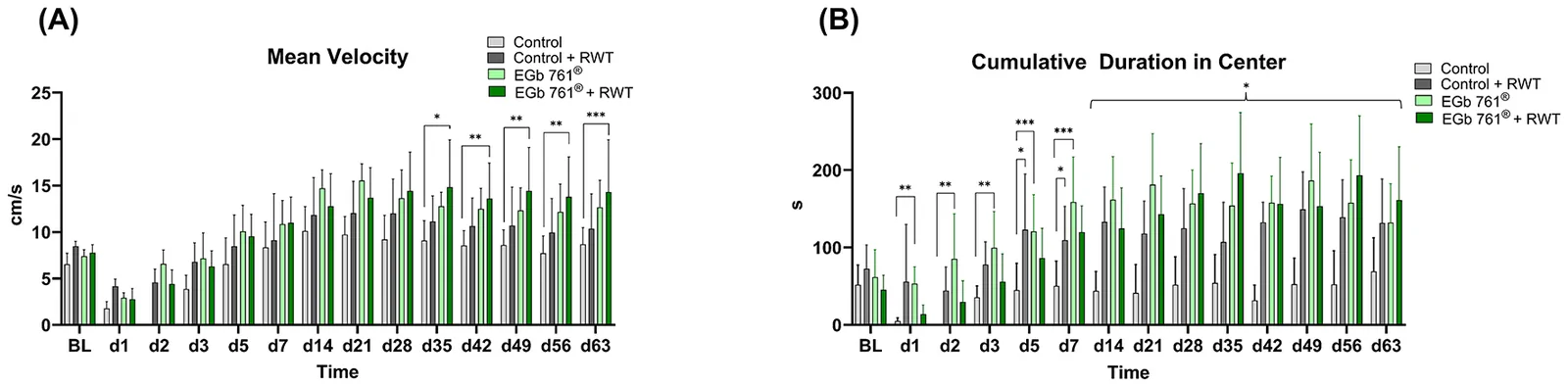
Quantitative locomotor parameters in the treatment groups. Effects of EGb 761^®^ and running wheel training (RWT) on locomotor velocity **(A)** and duration of stay in the center of the open field **(B)**, assessed using linear mixed-effects models (Time x EGb 761^®^ x RWT, animal as random effect). *Post-hoc* comparisons of each treatment group against Control (Control food, No RWT) were performed at each time point (Dunnett-type contrasts) with additional Holm correction across all time points. Bracket from d14 to d63: EGb 761^®^, EGb 761 ^®^ + RWT and Control + RWT significantly differed from Control at all time points within this range (*p* < 0.05). The values are presented as means with standard deviation. BL = baseline, control = control food, d = day * *p* < 0.05, ** *p* < 0.01, *** *p* < 0.001.

Cumulative duration in center showed a similar pattern and was influenced by Time, Food, RWT, Time x Food, Time x RWT and Food x RWT (Supplementary Table 4). Significant group differences between all groups and and control food could be found from d14 to d63 post cUL (Figure 3B).

### *In vivo* changes of whole-brain and regional synaptic density visualized by [^18^F]UCB-H μPET

Compared to baseline before cUL, voxel-wise analysis revealed a loss of synaptic density after cUL in the vestibular nuclei with the peak at d63 in all treatment groups except the EGb 761^®^ group (Figure 4A). The control food group showed the earliest onset (at day 21) and the most pronounced loss of synaptic density at d63 post cUL in both vestibular nuclei and the vestibulocerebellum. Treatment with EGb 761^®^ + RWT and control food + RWT diminished the extent of synaptic loss in the vestibular nuclei at 9 weeks post cUL. In the EGb 761^®^ group synaptic density at 63 day post cUL was at baseline level, indicating an almost complete recovery from cUL-induced synaptic loss by that time.

**Figure 4:**
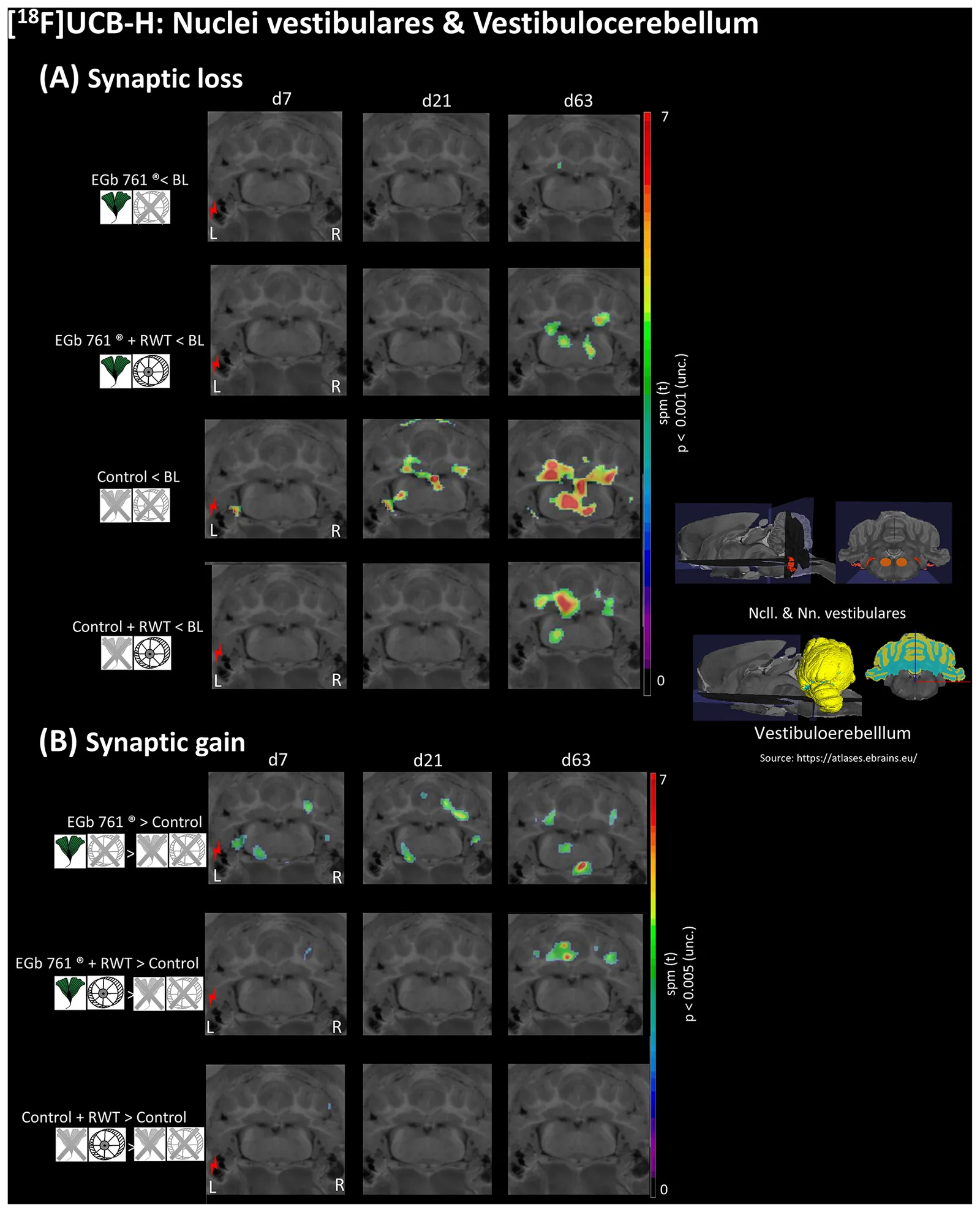
Voxel-wise analysis of synaptic density change across time points and groups. **(A)** Decrease of synaptic density in comparison to the baseline (BL, prior to cUL) for EGb 761^®^, EGb 761^®^ + RWT, control food and control food + RWT groups. **(B)** Increase of synaptic density relative to the control food group for EGb 761^®^, EGb 761^®^ + RWT and control food + RWT groups in the vestibular nuclei and vestibulocerebellum. T-values were obtained from voxel-wise t-test comparisons of each group to their baseline **(A)** or comparisons of each group to the control food group **(B)**. Significance thresholds of *p* < 0.001 (unc.) and *p* < 0.005 (unc.) were selected and the significant changes were shown with an extended threshold of k = 20 voxels. Control = control food, d = day, RWT = running wheel training.

Compared to the control food group, a relative gain of synaptic density was found in the vestibular nuclei and vestibulocerebellum in the EGb 761^®^ group and in the vestibulocerebellum in the EGb 761^®^ + RWT group, but not in the control food + RWT group (Figure 4B). Synaptic reoccupation in the vestibulocerebellum was visible in the EGb 761^®^ group after d7 and in the EGb 761^®^ + RWT group at d63 after cUL.

A relative gain of synaptic density was detected in the somatosensory cortices in treatment groups 2–4 at week 1 post cUL compared to the control food group (Figure 5A). In the EGb 761^®^ group enhanced synaptic density in these brain regions was found at week 3 post cUL, in the EGb 761^®^ + RWT group up to week 9 post cUL.

**Figure 5:**
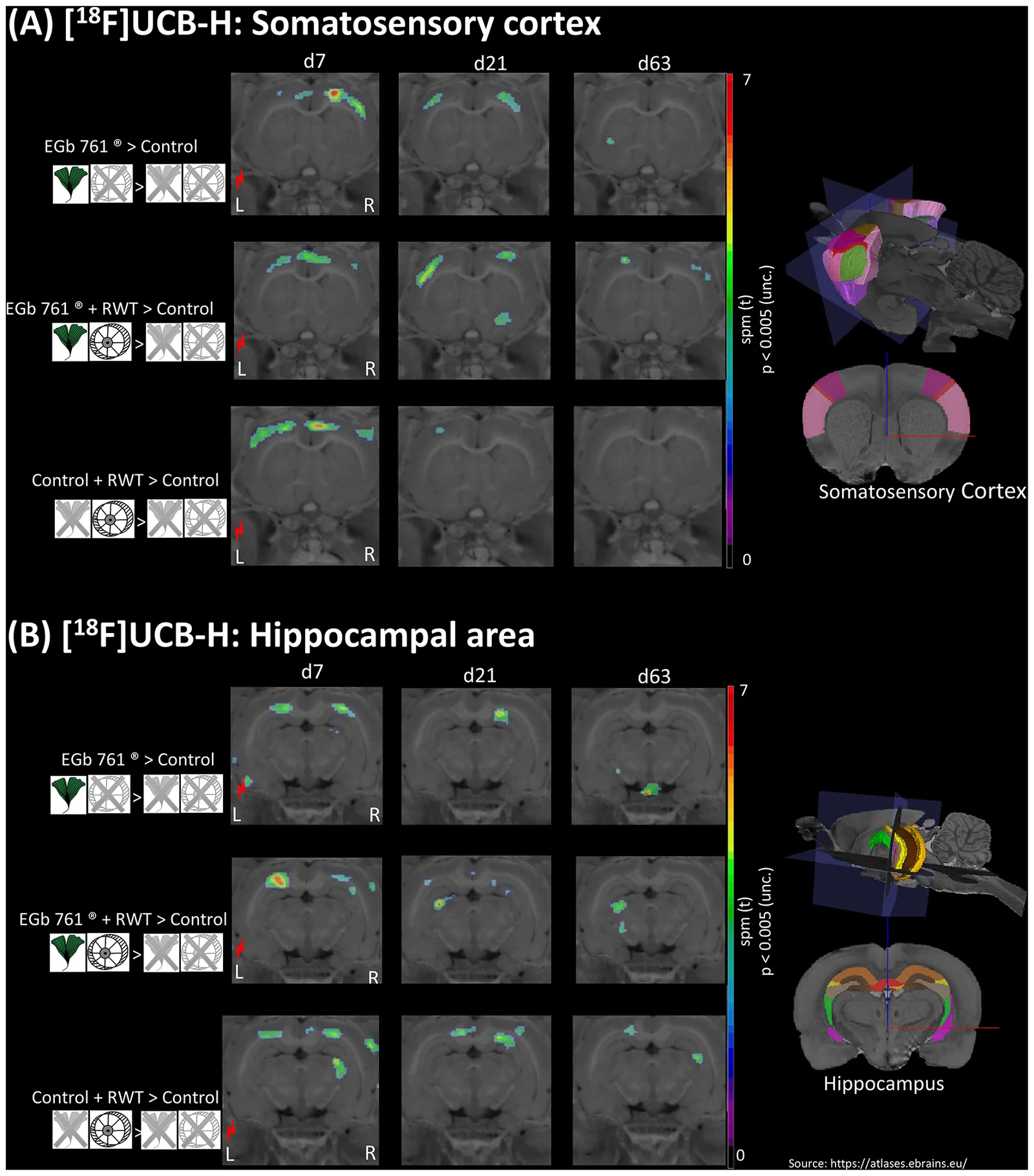
Increase of synaptic density in somatosensory cortices **(A)** and hippocampal area **(B)** in the EGb 761^®^, EGb 761^®^ + RWT and control food + RWT groups relative to the control food group. T-values were obtained from voxel-wise t-tests comparing each group to the control food group at the respective time point. A significance threshold of *p* < 0.005 (unc.) was selected and the significant changes were shown with an extended threshold of k = 20 voxels. Control = control food, d = day, RWT = running wheel training.

Direct comparisons between treatment groups 2–4 and the control food group also revealed a relative synaptic gain in the hippocampal area at d7 and d21 post cUL, which was strongest in the EGb 761 ^®^ + RWT group at d7 post cUL (Figure 5B).

Mixed-effects models of [^18^F]UCB-H uptake in segmented brain regions revealed a statistically significant impact of Time, the interaction of EGb 761^®^ x RWT and Time x EGb 761^®^ x RWT on the change of synaptic density in the vestibular nuclei (see Supplementary Table 5). In the motor cortex, Time and its interaction with EGb 761^®^ (Time x EGb 761^®^) and Time x EGb 761^®^ x RWT had a significant impact.

Relative synaptic change in selected brain regions for d7, d21 and d63 in comparison to baseline confirmed the results of voxel-wise analysis. The greatest synaptic loss was visible in the vestibular nuclei at d63 for the control food group (-20.37 ± 5.18%), with a significant difference to control food + RWT (-10.32 ± 7.41%, *p* = 0.022) and EGb 761^®^ group (-7.59 ± 7.0%, *p* < 0.001) (Figure 6A). In contrast, the motor cortex displayed the highest relative gain of synaptic density at d63 in the control food group (6.60 ± 3.13%), with a statistically significant difference to EGb 761^®^ + RWT (1.84% ± 4.19, *p* = 0.01) and EGb 761^®^ group (0.19% ± 2.82, *p* < 0.001) (Figure 6B).

**Figure 6:**
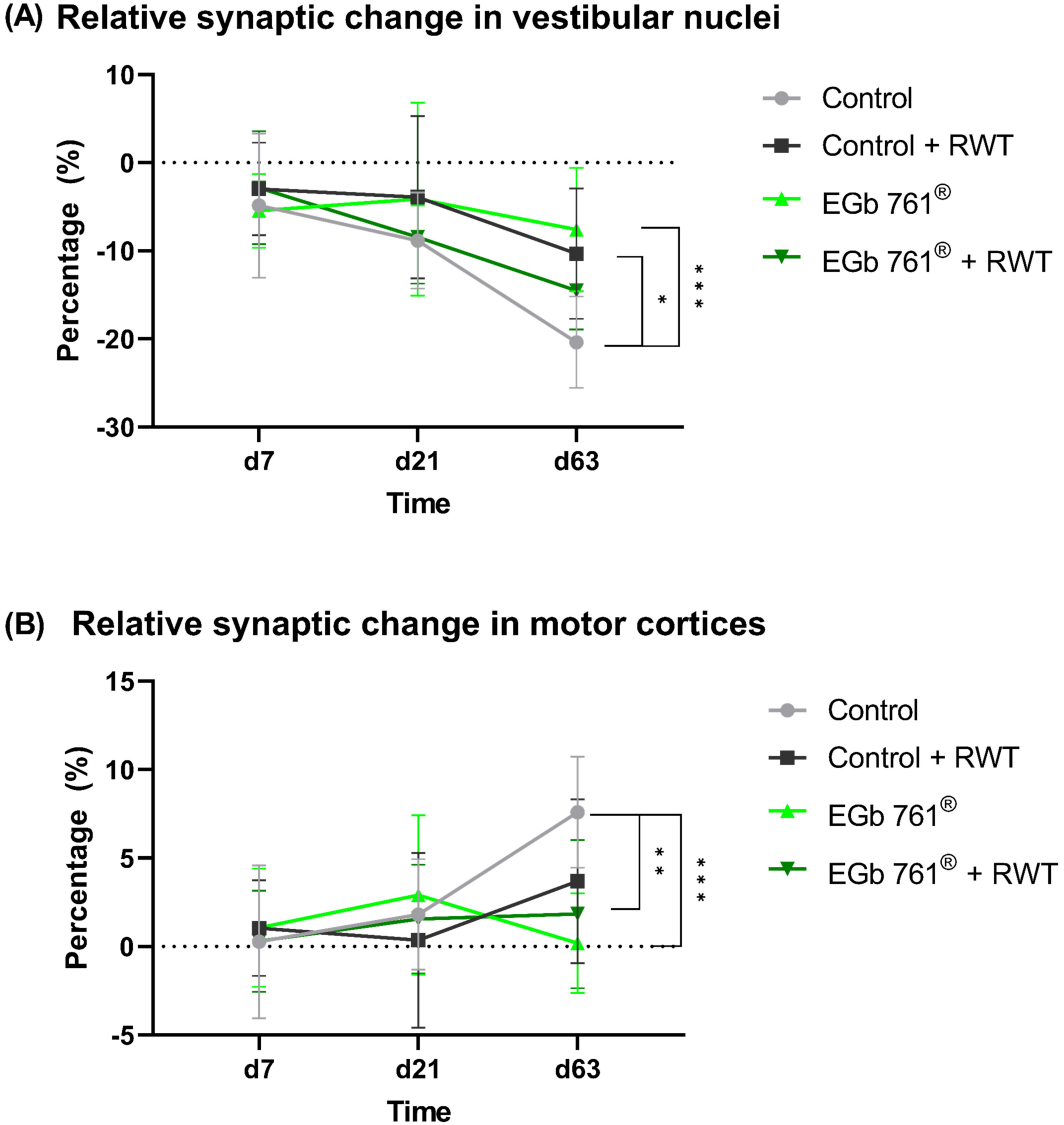
Changes of synaptic density relative to baseline in the vestibular nuclei **(A)** and motor cortices **(B)**. Synaptic density was assessed longitudinally via [^18^F]UCB-H PET and analyzed using a three-way repeated-measures mixed-effects model (Time x EGb 761^®^ x RWT, REML). Values are presented as mean ± standard deviation, individual values are marked as dots. Control = control food, d = day, RWT = running wheel training, * *p* < 0.05, ** *p* < 0.01, *** *p* < 0.001.

## Discussion

Fundamental research in preclinical vestibular lesion models only recently gained momemtum to identify the biological and functional effects underlying commonly applied clinical treatment principles in acute unilateral vestibulopathy such as RWT training and drugs targeting neuroplasticity (33–35). Following this backtranslational approach, the current study investigated the effects of voluntary running wheel training (RWT) and treatment with the Ginkgo biloba extract EGb 761^®^ on functional recovery and structural whole-brain reorganization after cUL in the rat. The major findings were:

1. The combination of EGb 761^®^ and RWT drove recovery of static vestibular deficits (head roll tilt, postural asymmetry) and improved locomotor behavior post cUL. This was confirmed by longitudinal mixed-effects modeling, which showed a significant Time x Food x RWT interaction for head hilt and postural asymmetry with a comparable trend for elevation tail rotation test. Nystagmus, in contrast, followed a purely time-dependent acute rise-and-resolution course without modulation by either treatment, consistent with its characteristically self-limiting nature.

2. Open-field behavior appeared to be influenced differently from the clinical vestibular scores. Whereas the clinical scores showed significant Time x EGb 761^®^ x RWT interactions, open-field parameters were primarily affected by main effects and lower-order interactions involving Time, EGb 761^®^, and RWT. In particular, EGb 761^®^ significantly influenced both mean velocity and cumulative duration in center, while RWT contributed to selected locomotor outcomes. These findings suggest that exploratory locomotor behavior and clinical vestibular recovery may be mediated by partly distinct processes and exhibit different sensitivities to pharmacological and rehabilitative interventions.

3. This behavioral pattern was mirrored on PET imaging: synaptic density in both the vestibular nuclei and motor cortex showed a significant Time x Food x RWT interaction, indicating that the combination treatment's behavioral benefit is accompanied by a corresponding structural correlate in vestibular and motor-related regions, rather than reflecting symptom masking alone. EGb 761^®^ alone most effectively reduced lesion-induced synaptic loss in the vestibular nuclei and vestibulocerebellum, while the EGb 761^®^ + RWT combination additionally increased synaptic density in the hippocampal formation. Synaptic density in somatosensory cortices increased in all treatment groups relative to control, potentially reflecting multisensory cortical recruitment during compensation, while more extensive vestibular nuclear synaptic loss was paralleled by greater synaptic gain in motor cortex, suggesting secondary adaptive motor cortical remodeling.

The current study confirms previous findings in animal models that EGb 761^®^ improves behavioral recovery of static and dynamic vestibular functions after a unilateral peripheral vestibular lesion at doses which equal those of standard treatment regimens in humans (6, 7, 36, 37). Furthermore, we could show for the first time in an animal model that the combination of EGb 761^®^ with voluntary running activity has additive benefits for behavioral compensation. More precisely, the recovery of postural asymmetry in the EGb 761^®^ + RWT group was completed by d35 post cUL, while mild postural asymmetry persisted in the other treatment groups until d63 post cUL. It is conceivable that EGb 761^®^ fosters the effect of voluntary locomotor training by enhancing adaptive neuroplasticity. Indeed, prospective clinical trials in patients with various peripheral and central vestibular disorders have also found a significant functional improvement of postural control and patients-reported outcomes, when vestibular rehabilitation was combined with EGb 761^®^ treatment (38–40). In addition, open-label real-world studies in patients with various vestibular disorders have documented an improvement of clinical and patient-reported outcomes with EGb 761^®^ administration (41).

Beyond the behavioral improvements demonstrated in this study, brain imaging of synaptic density provides important insights into the neural mechanisms underlying accelerated vestibular compensation with EGb 761^®^ and RWT treatment. Previous *in vitro* research indicated direct and indirect effects of EGb 761^®^ on synaptic plasticity mostly within the hippocampal formation in aging and neurodegeneration models: EGb 761^®^ prevents the downregulation of critical synaptic proteins such as synptophysin in models of neuronal damage (42), enhances long-term potentiation (LTP) in hippocampal slices of aged mice, which is a key cellular process for synaptic memory formation (43) and increases neurite outgrouth in neuronal cell lines (44). The current study markedly adds to this evidence, as it 1) presents a whole-brain visualization of dynamic synaptic density changes induced by EGb 761^®^ in the living animal for the first time, and 2) indicates a predominant effect of EGb 761^®^ on synaptic plasticity in regions and networks, which are known to be involved in central compensation of the peripheral vestibular damage, such as the vestibular nuclei, somatosensory and motor cortices and the hippocampal formation (45, 46).

Importantly, EGb 761^®^ alleviated the synaptic loss in the vestibular nuclei after cUL by about two thirds compared to the control group at 9 weeks post cUL. Similar findings were reported in an immunohistochemical study in the unilateral neurectomy cat model, which showed an accelerated synaptic reoccupation to baseline level at 3 months in the EGb 761^®^ vs. 5 months in the control group (6). In this previous study, the percentual loss of synaptic density in the control group was quantified as 35 % at week 1 and 14 % at week 3 post surgery, which ranges higher than in the present study. In this respect it needs to be considered that a vestibular neurectomy leads to a complete vestibular deafferentation compared to an incomplete lesion in cUL and therefore results in more intense synaptic loss (47). On the other hand, immunhistochemical methods may be more sensitive to depict changes of synaptic density compared to *in vivo* imaging methods such as [^18^F]UCB-H μPET. Nevertheless, both modalities point toward an acceleration of synaptic reoccupation by EGb 761^®^ in the first 3 months after unilateral peripheral vestibular lesions. The time constant for synaptic reoccupation in the vestibular nuclei is longer than for behavioral recovery of static vestibular symptoms, which may point to additional plasticity mechanisms augmented by EGb 761^®^ during early vestibular compensation. Indeed, it was reported previously that EGb 761^®^ can induce changes in cerebral energy metabolism already in the first week after cUL (7).

Voluntary running activity also reduced synaptic loss in the vestibular nuclei, albeit not to the extend that was found during EGb 761^®^ treatment. Previous studies have shown that sensorimotor rehabilitation can promote adaptive plasticity within deafferented vestibular networks (34). Furthermore, increased use of proprioceptive inputs after vestibular lesions has been proposed as a compensatory mechanism contributing to functional recovery (48–50). While the present study cannot identify the cellular mechanisms underlying these effects, the observed preservation of SV2A-derived PET signal is consistent with adaptive neuroplastic changes during vestibular compensation.

Beyond vestibular brainstem circuits, the combined EGb 761^®^ + RWT treatment was associated with increased synaptic density in the hippocampal formation. Previous studies have shown that voluntary running activity can promote hippocampal plasticity, whereas EGb 761^®^ has been reported to enhance neuroprotective and plasticity-related pathways (21, 51–53).

We further found differential effects of EGb 761^®^ and RWT on synaptic density in cortical somatosensory and motor networks. Generally all specific treatments increased synaptic density in the somatosensory cortices, which may speak for multisensory reweighting and adaption during successful vestibular compensation. In accordance, an immediate expansion of the cortical neuron cutaneous receptive fields and partial dedifferentiation of somatotopic maps was found after unilateral vestibular neurectomy in the rat (54). Synaptic density in the motor cortices also increased until 9 weeks post cUL in the control group. This effect was inversed by a faster behavioral recovery and more complete synaptic reoccupation in the vestibular nuclei especially in the EGb 761^®^ and EGb 761^®^ + RWT groups. One could hypothesize that structural remodeling in the motor cortices is a rather secondary adaptive mechanism to cope with persistent vestibular asymmetry. In accordance, we have shown earlier that the motor cortex functional connectivity is highest during the peak of vestibular asymmetry and normalizes to baseline level in case of behavioral recovery (45).

### Limitations

Synaptic density was assessed exclusively via longitudinal [^18^F]UCB-H PET without histological validation (e.g., SV2A, synaptophysin, PSD95); findings should therefore be interpreted as imaging-based estimates rather than direct evidence of synaptic remodeling. Whole-brain voxel-wise analyses used uncorrected thresholds and are exploratory; principal conclusions rely on the predefined regional and behavioral analyses. EGb 761^®^ was administered *ad libitum* via chow without quantification of individual intake, though plasma bilobalide/ginkgolide levels were previously confirmed under an identical protocol (7). Only male rats were studied, limiting generalizability to females. Finally, the interaction between training intensity and pharmacological modulation, and the differentiation between excitatory and inhibitory synaptic changes, remain open questions for future work (55, 56).

In conclusion, this study reinforces the value of multimodal therapeutic strategies for vestibular disorders. By combining the neuroprotective and synaptogenic effects of EGb 761^®^ with the activity-dependent plasticity induced by voluntary running wheel training, the intervention appears to target complementary mechanisms, resulting in more efficient and robust recovery. These findings support the development of individualized rehabilitation protocols in neurotology that integrate both pharmacological and behavioral components to maximize functional outcomes.

## Data Availability

The raw data supporting the conclusions of this article will be made available by the authors, without undue reservation.
