# Supplementary material for: Voluntary physical activity and pharmacological treatment with the Ginkgo biloba extract EGb 761 alleviate regional alterations in cerebral synaptic density and improve behavioral recovery after unilateral labyrinthectomy in the rat
Source: Front Neurol. 2026 Oct 23;17:1937612. doi: 10.3389/fneur.2026.1937612 (PMC13739543; doi:10.3389/fneur.2026.1937612)
Supplement: Supplementary file 1 [file Data_Sheet_1.pdf]

Supplementary Material

Supplementary Table 1: Number of animals included int the [^{18}F]UCB-H PET analyses for each experimental group and time point. BL = Baseline, d = day.

Animal numbers [^{18}F]UCB-H PET				
	BL	d7	d21	d63
Control	n = 6	n = 7	n = 8	n = 5
Control + RWT	n = 8	n = 8	n = 6	n = 8
EGb 761®	n = 8	n = 7	n = 6	n = 6
EGb 761® + RWT	n = 8	n = 8	n = 8	n = 8

Supplementary Table 2: Number of animals included int the behavioral analyses for each experimental group and time point. BL = Baseline, d = day.

Behavior (Clinical Scoring + Open Field)														
	BL	d1	d2	d3	d5	d7	d14	d21	d28	d35	d42	d49	d56	d63
Control	n = 8	n = 8	n = 8	n = 8	n = 8	n = 8	n = 8	n = 8	n = 8	n = 8	n = 8	n = 8	n = 8	n = 8
Control + RWT	n = 8	n = 8	n = 8	n = 8	n = 8	n = 8	n = 8	n = 8	n = 8	n = 8	n = 8	n = 8	n = 8	n = 8
EGb 761®	n = 8	n = 7	n = 7	n = 7	n = 7	n = 7	n = 7	n = 7	n = 7	n = 7	n = 7	n = 7	n = 7	n = 7
EGb 761® + RWT	n = 8	n = 8	n = 8	n = 8	n = 8	n = 8	n = 8	n = 8	n = 8	n = 8	n = 8	n = 8	n = 8	n = 8

Supplementary Table 3: Likelihood ratio test results (χ^2, df, p-value) for fixed effects and interactions from the cumulative link mixed models fitted to each behavioral clinical scoring domain (Time, Food, RWT, and their interactions; Animal ID as random intercept). Model specification differed per outcome based on data structure; see Methods for details.

**Behavior**	**Term**	**χ^2**	**df**	**p-value**
Head Tilt	Time	67.485	2	<0.001
Head Tilt	Food	6.3481	1	0.012
Head Tilt	RWT	12.211	1	<0.001
Head Tilt	Time x Food	12.138	2	0.002
Head Tilt	Time x RWT	14.692	2	<0.001
Head Tilt	Food x RWT	0.24	1	0.625
Head Tilt	Time x Food x RWT	9.368	2	0.009
Postural Asymmetry	Phase	220.438	1	<0.001
Postural Asymmetry	Food	15.409	1	<0.001
Postural Asymmetry	RWT	12.173	1	<0.001
Postural Asymmetry	Phase x Food	1.0942	1	0.296
Postural Asymmetry	Phase x RWT	0.972	1	0.324
Postural Asymmetry	Food x RWT	5.723	1	0.017
Postural Asymmetry	Phase x Food x RWT	17	1	<0.001
Nystagmus	Time	73.729	1	<0.001
Nystagmus	Food	0.996	1	0.318
Nystagmus	RWT	0.273	1	0.602
Nystagmus	Time x RWT	0.289	1	0.591
Elevation Tail Rotation	Time	18.746	2	<0.001
Elevation Tail Rotation	Food	0.367	1	0.545
Elevation Tail Rotation	RWT	1.428	1	0.232
Elevation Tail Rotation	Time x Food	2.771	2	0.250
Elevation Tail Rotation	Time x RWT	1.483	2	0.476
Elevation Tail Rotation	Food x RWT	1.441	1	0.230
Elevation Tail Rotation	Time x Food x RWT	5.649	2	0.059
Nystagmus	Time x Food	NA	NA	NA
Nystagmus	Food x RWT	NA	NA	NA

Supplementary Table 4: Likelihood ratio test results (χ^2, df, p-value) for fixed effects and interactions from the cumulative link mixed models fitted to each Open Field domain (Time, Food, RWT, and their interactions; Animal ID as random intercept). Model specification differed per outcome based on data structure; see Methods for details.

**Outcome**	**Term**	**χ^2**	**df**	**p-value**
Mean Velocity	Time	132,84	1	<0.001
Mean Velocity	Food	9,994	1	0.002
Mean Velocity	RWT	1,885	1	0.170
Mean Velocity	Time x Food	16,697	1	<0.001
Mean Velocity	Time x RWT	4,546	1	0.033
Mean Velocity	Food x RWT	1,364	1	0.243
Mean Velocity	Time x Food x RWT	1,633	1	0.201
Cumulative Duration in Center	Time	109,16	1	<0.001
Cumulative Duration in Center	Food	13,496	1	<0.001
Cumulative Duration in Center	RWT	4,401	1	0.036
Cumulative Duration in Center	Time x Food	22,119	1	<0.001
Cumulative Duration in Center	Time x RWT	18,317	1	<0.001
Cumulative Duration in Center	Food x RWT	15,006	1	<0.001
Cumulative Duration in Center	Time x Food x RWT	0,755	1	0.385

Supplementary Table 5: Results of the three-way repeated-measures mixed-effects model (Time x EGb 761® x RWT, REML) for [^{18}F]UCB-H PET-derived synaptic density in the vestibular nuclei and motor cortices. Fixed effects and their interactions are shown with degrees of freedom (df), F-statistics, and p-values.

**Nuclei vestibulares**	**df**	**F**	**p-value**
Time	2	35.56	< 0.001
EGb 761®	1	1.448	0.236
RWT	1	1.123	0.298
Time x EGb 761®	2	2.427	0.102
Time x RWT	2	0.5485	0.582
EGb 761® x RWT	1	11.30	0.002
Time x EGb 761® x RWT	2	7.956	0.001
**Motor cortices**	**df**	**F**	**p-value**
Time	2	7.288	0.001
EGb 761®	1	2.987	0.092
RWT	1	2.150	0.153
Time x EGb 761®	2	9.288	< 0.001
Time x RWT	2	0.6261	0.54
EGb 761® x RWT	1	1.733	0.196
Time x EGb 761® x RWT	2	3.915	0.028

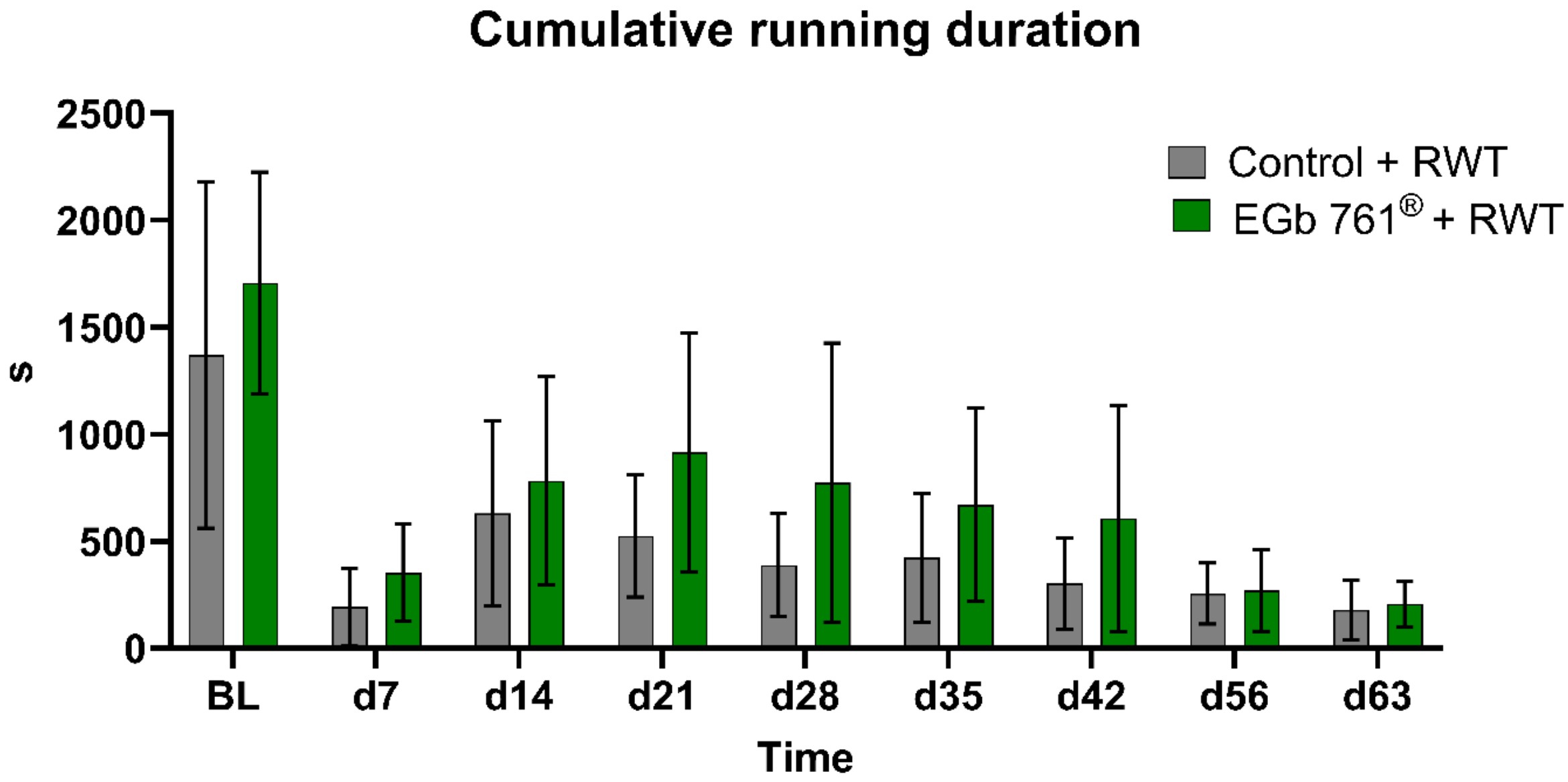

Supplementary Figure 1: Cumulative duration of running wheel use per week in the EGb 761® and control food groups. The values are presented as means with standard deviation. BL = baseline, d = day.
